# Norovirus Changes Susceptibility to Type 1 Diabetes by Altering Intestinal Microbiota and Immune Cell Functions

**DOI:** 10.3389/fimmu.2019.02654

**Published:** 2019-11-12

**Authors:** James A. Pearson, Ningwen Tai, Dilrukshi K. Ekanayake-Alper, Jian Peng, Youjia Hu, Karl Hager, Susan Compton, F. Susan Wong, Peter C. Smith, Li Wen

**Affiliations:** ^1^Endocrinology, Internal Medicine, School of Medicine, Yale University, New Haven, CT, United States; ^2^Colombia Center for Transplant Immunology and Institute of Comparative Medicine, Columbia University Medical Center, Colombia University, New York, NY, United States; ^3^Department of Comparative Medicine, School of Medicine, Yale University, New Haven, CT, United States; ^4^Department of Lab Medicine, School of Medicine, Yale University, New Haven, CT, United States; ^5^Diabetes Research Group, Division of Infection and Immunity, School of Medicine, Cardiff University, Cardiff, United Kingdom

**Keywords:** norovirus, type 1 diabetes, non-obese diabetic mice, gut microbiota, Tuft cells, Treg

## Abstract

Environmental factors contribute to Type 1 diabetes (T1D) susceptibility. The gut microbiome, which includes bacteria, viruses, and fungi, contributes to this environmental influence, and can induce immunological changes. The gut viral component of the microbiome, related to T1D has mostly focused on coxsackieviruses and rotavirus. The role of norovirus, another common enteric virus, in susceptibility to T1D was hitherto unknown. Norovirus is highly infectious and encountered by many children. We studied the mouse norovirus 4 (MNV4), related to human noroviruses, in the Non-obese diabetic (NOD) mouse model, to determine its role in influencing susceptibility to T1D. We infected MNV-free NOD mice with MNV4 by exposing the mice to MNV4-positive bedding from an endemically-infected mouse colony to mimic a natural infection. Control MNV-free NOD mice were exposed to MNV-free bedding from the same colony. Interestingly, MNV4 infection protected NOD mice from the development of T1D and was associated with an expansion of Tregs and reduced proinflammatory T cells. We also found MNV4 significantly modified the gut commensal bacteria composition, promoting increased α-diversity and Firmicutes/Bacteroidetes ratio. To elucidate whether T1D protection was directly related to MNV4, or indirectly through modulating gut microbiota, we colonized germ-free (GF) NOD mice with the MNV4-containing or non-MNV4-containing viral filtrate, isolated from filtered fecal material. We found that MNV4 induced significant changes in mucosal immunity, including altered Tuft cell markers, cytokine secretion, antiviral immune signaling markers, and the concentration of mucosal antibodies. Systemically, MNV4-infection altered the immune cells including B cell subsets, macrophages and T cells, and especially induced an increase in Treg number and function. Furthermore, *in vitro* primary exposure of the norovirus filtrate to naïve splenocytes identified significant increases in the proportion of activated and CTLA4-expressing Tregs. Our data provide novel knowledge that norovirus can protect NOD mice from T1D development by inducing the expansion of Tregs and reducing inflammatory T cells. Our study also highlights the importance of distinguishing the mucosal immunity mediated by bacteria from that by enteric viruses.

## Introduction

Type 1 diabetes (T1D) is a T cell mediated autoimmune disease resulting from the destruction of insulin-producing pancreatic β-cells. The incidence of T1D is increasing worldwide, at a rate too fast to be associated with genetic changes alone. Increasing evidence suggests that environmental factors can contribute to the risk of T1D development. Studies in both animal models and humans have identified changes in the bacterial component of the gut microbiota composition in individuals with T1D or “at-risk” compared to controls (1–11). In addition, enteric viruses, such as the Coxsackieviruses and Rotavirus have been associated with the development of T1D in both humans and mouse models (12–21). Interestingly, a coxsackievirus B vaccine has shown to protect Non-obese Diabetic (NOD) mice from the development of virus-induced diabetes (22) and a recent human study suggests that rotavirus vaccination of infants has contributed to the decreased incidence of T1D in Australian children (23). However, little is known about other viruses including norovirus in relation to T1D development.

Norovirus, a common enteric virus belonging to the family *Caliciviridae*, is highly contagious and responsible for the majority of non-bacterial gastroenteritis outbreaks, especially in the winter, and infections have often been referred to by the non-scientific name “stomach flu” (24). In most individuals, norovirus infections are short-lived but the infection can have severe complications in young children, the elderly and immunocompromised individuals (25–27). Transmission occurs by the fecal-oral route, either through direct contact with infected individuals or indirectly through exposure to contaminated food or water, and by infectious aerosols generated by vomiting (28–31). Given that the majority of individuals diagnosed with T1D are children, and the incidence appears to peak in winter (32) when it is also high season for norovirus infection, it is extremely important to investigate the role of norovirus in mediating susceptibility to T1D. It is likely norovirus infection is an additional stressor of the immune system prior to diagnosis and is not the cause of T1D, as many people infected with norovirus do not develop T1D.

Murine norovirus (MNV), a non-enveloped single-stranded RNA virus related to human noroviruses, is often used to study the role of norovirus infections in mouse models. MNV was first described following the investigation of high mortality in Rag/Stat-1 double-deficient mice (33). MNV has been shown to alter susceptibility to inflammatory bowel disease (IBD) and food allergies (34–36), by altering other components of the gut microbiota composition and gut immunity. Furthermore, the bacterial components of gut microbiota also affect the host susceptibility to norovirus infection, supported by the observation that antibiotic treatment prevented norovirus persistence due to changing the gut bacterial composition (37). Thus, MNV can alter both gut microbiota composition and immune responses of the host.

Our previous studies and those of other investigators have demonstrated that susceptibility to T1D development in NOD mice depends on immune signaling in response to the microbiota (8, 9, 38–41) and to coxsackievirus (42, 43) or rotavirus (17, 19). However, it is unknown if MNV alters the susceptibility to T1D development in NOD mice. We hypothesize that MNV infection will affect T1D susceptibility in NOD mice by changing gut commensal bacteria and hence local, as well as systemic, immune responses of the hosts. The current study was to test this hypothesis and to fill our knowledge gap regarding the role of norovirus infection in T1D development.

## Materials and Methods

### Mice

Specific pathogen-free (SPF) NOD mice were purchased from the Jackson Laboratory and housed under SPF conditions in individually-ventilated filter-topped cages at the Yale Animal Resource Center (YARC). These mice were reported free of murine norovirus, ectromelia virus, murine rotavirus, lymphocytic choriomeningitis virus, mouse hepatitis virus, mouse parvovirus, minute virus of mice, pneumonia virus of mice, reovirus, Sendai virus, *Mycoplasma pulmonis, Helicobacter* spp., pinworms, fur mites, and opportunistic bacteria (https://www.jax.org/strain/001976). Germ-free (GF) NOD mouse breeders were generously provided by Alexander Chervonsky (University of Chicago, USA) and have been bred and maintained at the gnotobiotic facility of YARC. All the mice received autoclaved food (Global 2018S, Envigo) and hyperchlorinated (4–6 ppm) water *ad libitum* and were maintained on 12-h light/dark cycles. The use of mice in this study was approved by the Institutional Animal Care and Use Committee at Yale University.

### MNV Detection and Infection

The SPF mouse housing room in which this study was conducted was screened for MNV infection by PCR of fecal samples. Fecal pellets were homogenized in PBS and DNA was isolated using a DNeasy kit (Qiagen) according to the manufacturer's instructions. PCR amplification was performed using a PCR Core kit (Roche) and primers specific for the MNV non-structural gene (see Supplementary Table 1). The strain of MNV identified by sequencing was consistent with MNV4. The MNV4-positive cage bedding was collected and introduced to the cages that housed MNV-free NOD mice (4–5 week of age). The cages had half the bedding replaced with autoclaved clean bedding, weekly. As a control, another set of MNV-free NOD mice were introduced to MNV-free bedding from different cages within the same housing room. To avoid cross-contamination, the control NOD mice were housed in a different room in the same facility. All the mice were screened by PCR for the presence of MNV4 in the fecal material and by an immuno-fluorescence assay for the presence of anti-MNV antibodies in the serum. Briefly, microscope slides were mounted with monolayers of MNV-infected RAW 264.7 cells, a mouse macrophage cell line. Serum samples (1:10 dilution) were added to the slides and the binding of MNV antibodies was detected with fluorescein-conjugated goat anti-mouse antisera. All MNV+ mice continued to actively shed virus throughout the study. Only mice exposed to MNV had anti-MNV antibodies in the serum. All control (MNV4-free) mice remained free of MNV infection.

### Diabetes Incidence

MNV-infected and control NOD mice were monitored for glycosuria weekly, for 25 weeks. Glycosuria was confirmed by two blood glucose measurements, 24-h apart, of over 250 mg/dl (>13.9 mmol/L).

### Histology

Pancreata from 12-week old MNV-free (control) and MNV-infected NOD female mice were formalin-fixed and embedded in paraffin. Tissues were stained with hematoxylin and eosin. Insulitis was scored under light microscopy. 150–200 islets from 4 to 5 mice were individually scored.

### 16s rRNA Sequencing of Gut Microbiota

Fecal samples were collected from MNV-free (control) and MNV-infected mice and resuspended in 300 μl TE buffer containing 0.5% SDS and 200 μg/ml Proteinase K. Bacterial DNA was extracted as previously described (44). The V4 region of the 16S rRNA gene was amplified from each DNA sample using a bar-coded, broadly conserved, bacterial forward, and reverse primer as previously published (8). Bacterial DNA samples were used for pyrosequencing with Ion Torrent PGM sequencing system (Life Technologies). The results were analyzed using QIIME 1.8. α-diversity, a measure of the number of bacteria, and β-diversity, a measure of the composition of the microbiota were both analyzed and β-diversity was plotted using a principal coordinate analysis (PCoA) plot.

### Flow Cytometry and Intracellular Staining

Immune cells were incubated with an Fc-blocking antibody at 4°C for 15 min. Post-incubation, cells were stained for surface markers using antibodies (conjugated with different fluorochrome) against CD4, CD8, CD11b, CD11c, CD19, CD21, CD23, CD39, CD69, CD86, CTLA4, CXCR3, KLRG1, TCRbeta, IgA, IgD, IgM, CCR6, CCR7, CCR9, and a viability dye (all from BioLegend), for 30 min at 4°C. For Treg staining, cells were stained for surface markers prior to fixation for 1 h at room temperature and subsequent permeabilization (buffers purchased from Tonbo Bioscience). The cells were then incubated with an Fc-blocking antibody at 4°C for 15 min prior to staining with anti-FoxP3 (eBioscience), incubated for 30 min, at 4°C; following by washing. For intracellular cytokine staining, cells were incubated at 37°C in the presence of PMA (Sigma), Ionomycin (Sigma) and Golgi Plug (BD) for 4 h prior to washing and surface staining as outlined above. After surface staining, cells were fixed (20 min, room temperature) and permeabilized (buffers purchased from Sigma) and incubated with an Fc-blocking antibody at 4°C for 15 min prior to staining with anti-cytokine antibodies (30 min, 4°C) and washing. Cells were analyzed on a BD LSR II flow cytometry followed by analysis using Flowjo software.

### Infection of Germ-Free NOD Mice

Fecal pellets were pooled from 6 individual 12-week old MNV4+ donors and resuspended in sterile PBS. Pellets were homogenized using a bead beater machine and large fecal materials were removed by centrifugation (500 rpm, 3 min). Supernatant was further centrifuged at high speed (13,000 rpm, 5 min) followed by filtration through a 0.22 μM filter (Millipore). This filtered supernatant was then divided into two portions, with half of the solution exposed to UV light (20 min, room temperature) to destroy the virus (virus –) and the other half left unexposed to UV light (virus +). GF NOD mice (~4 weeks-old) were gavaged with 200 μl of the UV-treated or non-UV treated solution. GF NOD mice were regularly assessed for viral presence in the fecal sample and antibodies in the serum, before and after inoculation, as described earlier. Mice were terminated 8-weeks after gavage for the study and the experimental design is shown in Supplementary Figure 1.

### qPCR

RNA was extracted from the distal small intestine of colonized GF NOD mice, using a Qiagen RNAeasy kit, prior to cDNA synthesis following the manufacturer's instructions (Bio-Rad). qPCR was performed using a qPCR cycler (iQ5; Bio-Rad Laboratories), according to the manufacturer's instructions with the specific primers listed in Supplementary Table 1. The relative gene expression was determined using the 2^−ΔΔCT^ method by normalization with GAPDH housekeeping gene.

### Antibody Measurements

Serum and cecal wash were collected from the mice studied at termination. Antibody concentrations were determined by ELISA, using the reagents purchased from Southern Biotech, following the protocol previously described (8). Samples were diluted (serum 1:50–1:100; cecal samples 1:2–1:10) before the antibody measurements. Antibody concentrations were converted based on each of the standard curves.

### Treg Suppression Assay

Treg cells were purified from spleen of colonized GF NOD mice using magnetic bead isolation kits (EasySep TM Mouse CD4+CD25+ Regulatory T cell isolation kit, Stemcell Technologies). BDC2.5 CD4 T cells were isolated from BDC2.5 T cell receptor (TCR) transgenic NOD mice by negative selection using hybridoma supernatants to deplete MHCII+ (10.12.16) antigen presenting cells (APCs) and CD8 T cells (TB105). Hybridoma supernatants were kindly provided by the late Charles Janeway Jr. (Yale University). Isolated Tregs were co-cultured with BDC2.5 CD4 T cells in a 1:2 (Treg:Teff) ratio in the presence of irradiated APCs and different concentrations of BDC2.5 mimotope peptide. The cells were cultured for 4 days prior to pulsing with ^3H^-thymidine, for a further 18 h. Data were presented as corrected counts per minute (Δcpm) after subtracting from background (Tregs + BDC2.5 CD4 T cells without antigenic peptide).

### *In vitro* Culture

Splenocytes from colonized GF NOD mice (2 × 10^6^) were cultured in the presence or absence of non-UV treated (MNV4-containing) fecal material, prepared as described above. Cells were stimulated for 16 h with the final 4 h in the presence of PMA, Ionomycin (both Sigma) and Golgi Plug (BD), prior to surface and intracellular staining as outlined above. Another set of cells was stimulated for 16 h with UV-treated and non-UV treated (MNV4-containing) fecal material for RNA isolation after removing the stimulators. The cultured cells were also used for T cell and APC isolation by negative selection using monoclonal antibody supernatants. For T cell isolation, 10.2.16, HB198 and N418 were used to remove MHC II+ cells by magnetic beads. For APC isolation, Y19 was used to remove Thy1+ T cells by complement. All the mAb supernatants were provided by the late Charles Janeway Jr. (Yale University).

### Adoptive Transfer

Splenocytes were cultured for 12 h, in the presence or absence of non-UV treated (MNV4-containing) fecal material, prepared as described above. After thorough washing, the stimulated splenocytes were adoptively transferred into Rag-deficient NOD mice (4 × 10^6^/donor). Tissues were harvested 1 week post-transfer.

## Results

### MNV4 Infection Protects NOD Mice From the Development of Type 1 Diabetes

**Figure 1:**
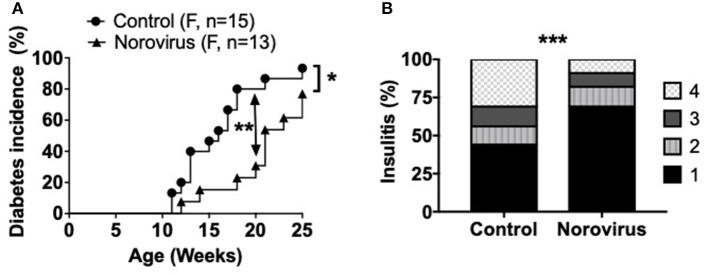
Norovirus infection protects NOD mice from the development of T1D. **(A)** Diabetes incidence was observed in MNV-infected (norovirus group) or MNV-negative (control) NOD mice, *n* = 13–15. Mice were monitored for glycosuria weekly for 25 weeks. Diabetes was confirmed by 2 blood glucose measurements, 24-h apart, of over 250 mg/dl (13.9 mmol/L). **(B)** 12-week old NOD mice infected or free of norovirus were harvested for their pancreata and assessed for insulitis by light microscopy, *n* = 5–6. Islets were graded using the following scale: 1: No insulitis, 2: >25% insulitis, 3: 25–50% insulitis, 4: >50% insulitis. Data were analyzed for significance using a log-rank test for survival at 20 and 25 weeks of age **(A)** or a chi-square test **(B)**. ^*^P < 0.05, ^**^P < 0.01, ^***^P < 0.001. Data are representative of one of two experiments.

To determine whether norovirus can alter the susceptibility of NOD mice to development of type 1 diabetes, MNV-free NOD mice were exposed at 4–5-weeks of age to bedding from either MNV- or MNV+ cages, of mice housed in the same facility. All MNV-infected mice were confirmed as virus positive by PCR in fecal samples, 2-weeks post-exposure, and the majority of the mice continued to shed virus throughout the study. All non-infected control mice remained virus-free. The mice from 10-weeks of age were tested weekly for glycosuria and followed longitudinally to determine the incidence of spontaneous diabetes in norovirus-infected and control mice. We found that norovirus exposure significantly delayed and reduced the incidence of diabetes development, compared to the control mice, by 25-weeks of age (Figure 1A). The delay was more striking at 14–20 weeks of age when only ~30% of infected mice developed diabetes by 20 weeks, compared to over 85% of control mice (*p* = 0.006). To determine, whether norovirus infection also reduced the immune cell infiltration in the pancreatic islets, pancreata from 12-week old pre-diabetic mice were studied for the severity of islet infiltration. In agreement with the diabetes incidence, we found norovirus-infected mice also had less insulitis compared to norovirus-free control NOD mice (Figure 1B).

### MNV4 Infection Promotes the Expansion of Regulatory T Cells and Reduces Inflammatory Cytokines and T Cell Activation

**Figure 2:**
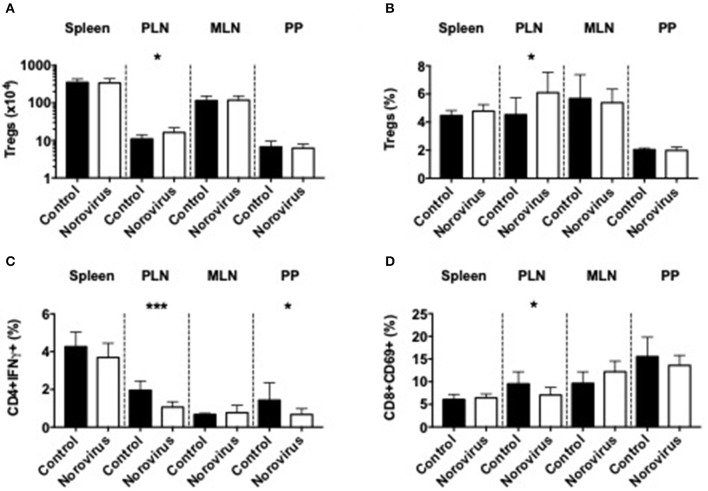
Norovirus infection expands Tregs and reduces inflammatory T cells. The number **(A)** and proportion **(B)** of CD4+FoxP3+ T cells were investigated from the spleen, pancreatic lymph node (PLN), mesenteric lymph node (MLN), and Peyer's patches (PP) of norovirus-free and norovirus-infected NOD mice. Tregs were gated on live single TCRbeta+CD4+CD8- T cells prior to FoxP3 gating. **(C)** Cells were stimulated for 4-h in the presence of PMA, Ionomycin, and Golgi Plug prior to surface and intracellular staining. IFNγ-secreting CD4 T cells were gated from live, single TCRbeta+CD4+CD8- T cells prior to gating on IFNγ. **(D)** The proportion of CD69+ CD8 T cells gated from live single TCRbeta+CD8+CD4- T cells prior to gating on CD69. Data were analyzed for significance using a Student's *T*-test. ^*^P < 0.05, ^***^P < 0.001. Data represents pooled data from two independent experiments; *n* = 9–11 mice.

As β-cell damage in T1D is an immune-mediated process we investigated changes of the immune system that could contribute to diabetes protection, in response to norovirus infection. We demonstrated that norovirus infection specifically enhanced the total number and proportion of regulatory T cells in the pancreatic draining lymph nodes (PLN) compared to uninfected control mice (Figures 2A,B and Supplementary Figure 2). We also evaluated the cytokine secretion profile from CD4 T cells and our results showed a significant reduction in IFNγ-secreting CD4 T cells, specifically in the PLN and Peyer's patches (PP), while the proportion of IL-10, TNFα, or IL-17a-secreting CD4 T cells remained unchanged (Figure 2C and Supplementary Figures 3A–D). Whilst we found no differences in CD4 T cell activation (CD69, Supplementary Figure 3E), we observed a significant reduction in CD8 T cell activation, particularly in the PLN (Figure 2D and Supplementary Figure 3F). We found no significant differences in IFNγ, TNFα, or IL-10 secretion from CD8 T cells (Supplementary Figures 3G–I). We also assessed changes in B cells, macrophages and dendritic cells in different lymphoid tissues and did not observe significant differences between the two groups (Supplementary Figure 4). Thus, the protection in the NOD mice infected with norovirus was associated with the expansion of Tregs and reduction of IFNγ-producing CD4 T cells and activation of CD8 T cells.

### MNV4 Infection Alters the Composition of Gut Commensal Bacteria

**Figure 3:**
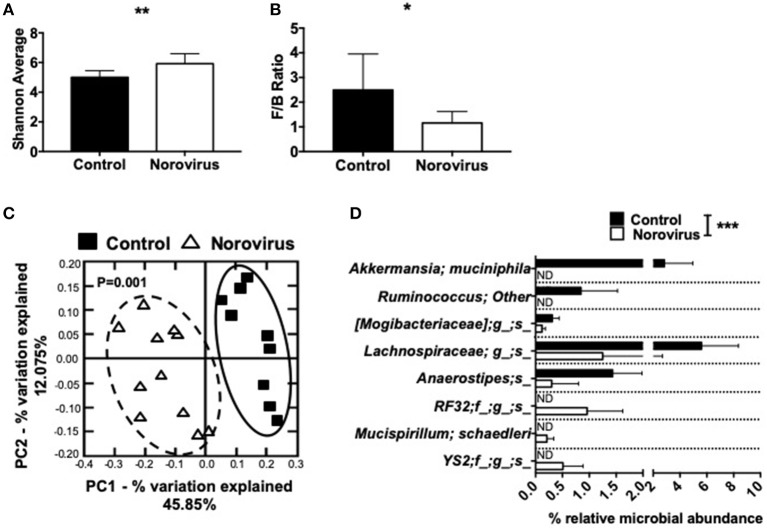
Norovirus infection alters the gut commensal bacterial composition in NOD mice. Fecal pellets were collected from 12-week old NOD mice that were MNV-free (control) or MNV-infected (norovirus). **(A)** α-diversity was assessed using the Shannon diversity index. **(B)** The Firmicutes/Bacteroidetes ratio were calculated from the phylogenetic data. **(C)** β-diversity was investigated using a principal component analysis plot, with significant species differences plotted **(D)**. Data were assessed for significance using a Student's *T*-test **(A,B)**, ANOSIM **(C)** or multiple *T*-test and FDR correction **(D)**. ^*^P < 0.05, ^**^P < 0.01, ^***^P < 0.001. Data shown are pooled from two experiments (*n* = 9–11 mice). ND, not detectable.

Our previous studies and those of others have shown that the changes in composition of gut commensal bacteria influence susceptibility to T1D in both NOD mice and humans (1, 3, 6, 8, 9, 11, 45). As norovirus is an intestinal virus and can alter the overall gut commensal bacteria composition in IBD (35), we investigated whether gut commensal bacterial composition was altered in MNV4-infected mice by 16S rRNA sequencing. We found that norovirus infection increased the intestinal microbial α-diversity and Firmicutes/Bacteroidetes ratio compared to the control mice that are more susceptible to diabetes (Figures 3A,B). Both of these changes have been reported to be associated with protection from the development of T1D in humans (1). To determine whether gut commensal bacterial composition was significantly different between uninfected control mice and norovirus-infected NOD mice, we conducted a principal component analysis of β-diversity. Our results revealed that the presence of norovirus significantly altered the composition of gut commensal bacteria (Figure 3C). Furthermore, we found significant increases in the relative abundance of *Akkermansia muciniphilia* and non-identifiable species of *Ruminococcus, Mogibacteriaceae, Lachnospiraceae*, and *Anaerostipes* in uninfected NOD mice compared to norovirus-infected NOD mice (Figure 3D). However, norovirus-infected mice had increased relative abundances of *RF32, Mucispirillum schaedleri* and *YS2* species compared to uninfected control mice. Together, our data demonstrate that MNV4 infection can alter the composition of gut commensal bacteria in NOD mice, suggesting a role in diabetes protection in the MNV4-infected NOD mice.

### MNV4 Infection of Germ Free (GF) NOD Mice Significantly Alters Intestinal Immunity

In the above experiments using SPF NOD mice, we infected the mice naturally with MNV+ bedding from the cages housing MNV-infected mice and the control mice had MNV- bedding. However, it was not clear whether diabetes protection and Treg cell expansion were the direct effect of norovirus or an indirect effect through virally-induced gut commensal bacterial changes. To elucidate the specific impact of norovirus on the immune system, we studied Germ-Free (GF) NOD mice. We infected GF NOD mice with pooled and filtered [removing the bacteria (46)] donor fecal material from mice infected with MNV4 (see Material & Methods and Supplementary Figure 1). As a control we UV-treated the same material to destroy the virus. All control mice remained negative for MNV4 (virus–) by PCR following colonization, and also negative for MNV-specific antibodies in serum, whereas the MNV4 infected mice (virus+) were positive for both virus in the feces and MNV-specific antibodies in the serum. Furthermore, we investigated the composition of gut microbiota from the infected mice but did not find significant changes between the virus-infected or non-virus-infected group (Supplementary Figure 5). Thus, the changes observed in the mice are predominantly associated with the presence/absence of norovirus.

**Figure 4:**
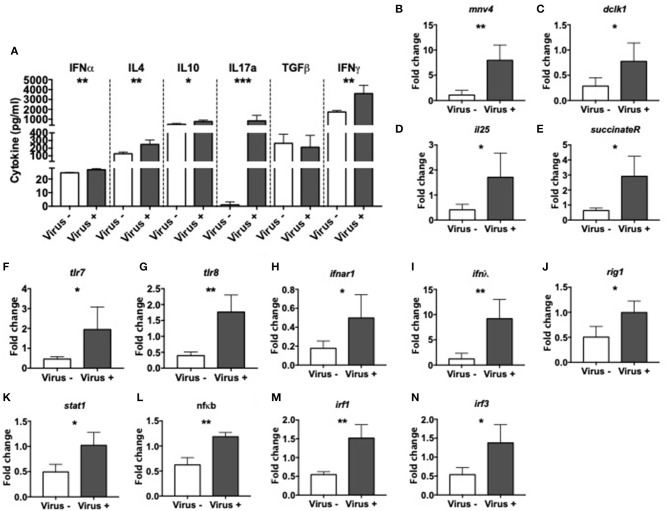
Altered intestinal immunity in norovirus infected NOD mice. GF NOD mice were orally gavaged at ~4 weeks of age with pooled and filtered norovirus-enriched fecal filtrate from norovirus-infected NOD mice at 12-weeks of age that was UV-treated (virus–) or non-UV-treated (virus+). Only mice gavaged with the non-UV-treated filtrate were infected with norovirus and developed anti-MNV antibodies. **(A)** Cytokines in cecal flushes from infected/non-infected mice were measured by ELISA. **(B–N)** RNA from the distal small intestine immediately adjacent to the cecum was extracted and equal concentrations of cDNA synthesized. cDNA was then subject to qPCR for MNV4, genes associated with Tuft cells [doublecortin-like kinase 1 (dclk1), IL25, succinate receptor 1; **C–E** respectively], with Toll-like receptor (TLR) genes (tlr7 and tlr8; **F,G** respectively) and antiviral immune signaling genes [Interferon α receptor 1 (ifnar1), interferon lamda (ifnλ), retinoic inducible gene I (rigI), signal transducer and activator of transcription 1 (stat1), nuclear factor kappa-light-chain-enhancer of activated B cells (nfκb), interferon regulatory factor (IRF) 1 and 3; **H–M** respectively]. The relative expressions of these genes were determined using the 2^−ΔΔCT^ method by normalization with GAPDH. Data were assessed for significance using a Student's *T*-test **(A–N)**. Data shown are representative of one of two experiments with *n* = 4 per group/experiment. ^*^P < 0.05, ^**^P < 0.01, ^***^P < 0.001.

To investigate the effect of MNV infection on intestinal immunity, we measured various cytokines in the cecal wash from virus– and virus+ ex-GF mice. We found significant increases in the concentration of IFNα, IL-4, IL-10, IL-17a, and IFNγ but no differences in TGFβ in virus+ mice compared to virus– mice (Figure 4A). Different MNV strains have different cell tropisms, and have been shown to infect both immune cells (47, 48) and specialized intestinal epithelial cells (IECs) called Tuft cells (49). To determine whether MNV4 were present in IECs, we isolated the IECs from the small intestine of ex-GF mice and detected MNV4 by qPCR. We found that the MNV4 gene was highly expressed in the mice exposed to MNV4 compared to the uninfected controls (Figure 4B). This suggests that IECs are most likely a target for MNV4 infection. Next, we assessed the Tuft cell-related gene expression in the small intestine of the ex-GF mice by qPCR, given MNV1 has been reported to infect Tuft cells (49). Interestingly, we found significant increases in Tuft cell-related genes including dlck1, IL-25, and succinate receptor (binding of which with succinate can activate Tuft cell responses) (50), in virus-infected mice compared to controls (Figures 4C–E). We also detected significant increases in the expression of single-stranded RNA-recognizing toll-like receptors (TLRs) 7 and 8, the IFNα receptor, IFNλ and antiviral signaling genes including Rig1, Stat1, NFκB, and IFN regulatory factors (IRF) 1 and 3 in virus+ mice when compared to virus- mice (Figures 4F–N). We did not observe any significant changes in the gene expression of tight junction (zonulin and claudin 2), the gut permeability markers; neither antimicrobial peptides or TLR3, which recognizes double-stranded RNA, between the two groups (Supplementary Figures 6A–H). Together, our data suggest that MNV significantly alters the intestinal immune responses resulting in altered cytokine secretion and activation of both Tuft cell and antiviral responses.

### MNV4 Infection of GF NOD Mice Significantly Alters APCs

**Figure 5:**
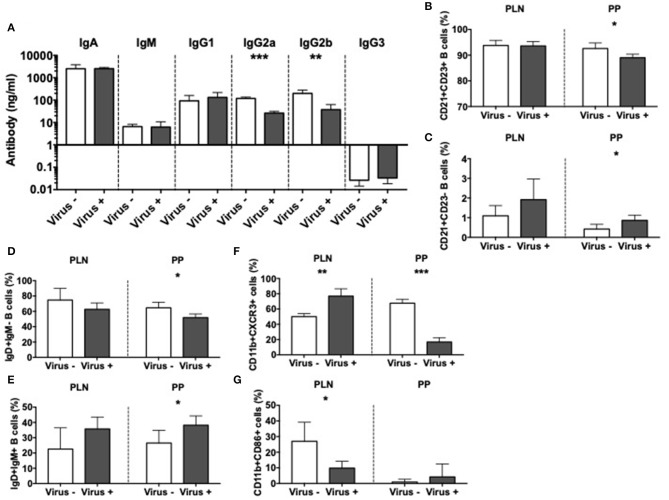
Altered APC immunity in norovirus infected NOD mice. GF NOD mice were orally gavaged at ~4 weeks of age with pooled and filtered norovirus-enriched fecal filtrate from norovirus-infected NOD mice at 12-weeks of age that was UV-treated (virus–) or non-UV-treated (virus+). Only mice gavaged with the non-UV-treated filtrate were infected with norovirus and developed anti-MNV antibodies. **(A)** Cecal antibody concentrations were determined by ELISA. **(B–G)** The proportion of cell subsets was determined by flow cytometric analysis. **(B,C)** The proportion of follicular (CD21+CD23+; **B**) and marginal zone (CD21+CD23-; **C**) B cells from the pancreatic lymph nodes (PLN) and Peyer's patches (PP) were gated from live, single CD19+TCRbeta- cells prior to gating on CD21/CD23. **(D,E)** The proportion of naïve (IgD+IgM-) and mature (IgD+IgM+) B cells were gated as in **B,C** prior to gating on IgD/IgM. **(F)** The proportion of CXCR3+ macrophages, gated from live, single CD19-TCRbeta-IA^g7^(MHCII)+CD11b+CD11c- cells prior to gating on CXCR3. **(G)** The proportion of CD86+ macrophages gated as in **F** prior to gating on CD86. All data were assessed for significance using a Student's *T*-test. Data shown are representative of one of two experiments with *n* = 4 per group/experiment. ^*^P < 0.05, ^**^P < 0.01, ^***^P < 0.001.

To further investigate the effect of MNV infection on immune cells, we focused on the antigen presenting cells (APCs). We first assessed the effect on mucosal B cells by evaluation of immunoglobulins in the gut lumen of infected and non-infected ex-GF mice. Our results revealed significant reductions in the concentrations of IgG2a and IgG2b subclasses in the intestine of MNV-infected mice compared to controls (Figure 5A); however, these changes were not present in the serum of the same mice (Supplementary Figure 7A). To probe if the mucosal antibody changes were due to changes of local B cells, we investigated the phenotype of B cells in PP and PLN. We found a reduced population of follicular CD21+CD23+ B cells but an increase in marginal zone CD21+CD23- B cells in the PP of virally-infected mice compared to the controls (Figures 5B,C and Supplementary Figure 7B). We also observed significant reductions in immature IgD+IgM- B cells, while IgD+IgM+ mature B cells were increased in virally-infected mice compared to the uninfected controls in the PP (Figures 5D,E and Supplementary Figure 7C). There was a similar trend in the PLN, although the changes were not statistically significant. There were no changes in IgD-IgM+ B cells or IgA+ B cells (Supplementary Figures 7D,E). Next, we investigated the effect of MNV infection on other APC populations. Our results showed the reduction of CXCR3+ macrophages in the PP but increased in the PLN of virally infected mice compared to the controls (Figure 5F and Supplementary Figure 8A). This suggested an enhanced recruitment of macrophages to the PLN, whereas the macrophage recruitment in PP was reduced; there were no changes in any other tissues studied (Supplementary Figure 8B). Interestingly, we observed significant reductions in CD86+ macrophages in the PLN, suggesting a reduced ability to activate the T cells in the PLN (Figure 5G and Supplementary Figure 8C). We did not find significant differences in CD11b+CD11c+ cells or CD11b-CD11c+ dendritic cells (Supplementary Figures 8D–G). Taking together, our data indicate that MNV4 infection predominantly alters mucosa-associated B cell responses as well as influences macrophage recruitment and costimulation locally.

### MNV4 Infection of GF NOD Mice Significantly Alters T Cells

**Figure 6:**
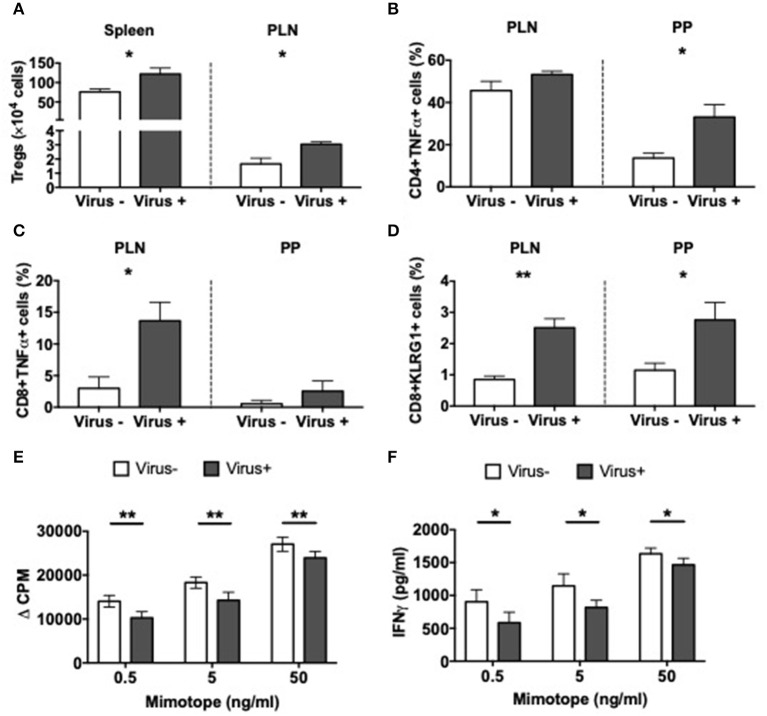
Altered T cell immunity in norovirus colonized NOD mice. GF NOD mice were orally gavaged at ~4 weeks of age with pooled and filtered norovirus-enriched fecal filtrate from norovirus-infected NOD mice at 12-weeks of age that was UV-treated (virus–) or non-UV-treated (virus+). Only mice gavaged with the non-UV-treated filtrate were infected with norovirus and developed anti-MNV antibodies. **(A)** The number of CD4+FoxP3+ Tregs were calculated from live single TCRbeta+CD4+CD8- T cells prior to FoxP3 gating in the spleen and pancreatic lymph node (PLN). **(B,C)** Cells were stimulated for 4-h in the presence of PMA, Ionomycin, and Golgi Plug prior to surface and intracellular staining. TNFα-secreting CD4 **(B)** or CD8 **(C)** T cells were gated from live single TCRbeta+ cells prior to CD4 or CD8 gating then subsequent gating on TNFα. Cells shown are from the PLN and Peyer's patches (PP). **(D)** The proportion of KLRG1+ CD8 T cells gated from CD19-TCRbeta+CD4-CD8+ T cells prior to gating on KLRG1+ cells in the PLN and PP. **(E)** Proliferation of BDC2.5 CD4+ T cells to mimotope peptide in the presence of Tregs (1:2) from virus- or virus+ mice. Background proliferation (APCs + BDC2.5 + Tregs without peptide) was subtracted from proliferation with peptide. Data are shown as change in counts per minute (ΔCPM). **(F)** IFNγ measured by ELISA from Treg suppression culture supernatants in **E**. All data were assessed for significance using a Student's *T*-test. Data shown are representative of one of two experiments with *n* = 4 per group/experiment. ^*^P < 0.05, ^**^P < 0.01.

Having identified significant differences in the APCs related to the viral infection, we asked if MNV infection also affects T cells. In line with our results in MNV-infected SPF mice, presented earlier (Figure 2A), we also discovered that MNV infection of GF mice significantly increased the number of Tregs in the spleen and PLN of ex-GF mice compared to the uninfected control ex-GF mice (Figure 6A). Interestingly the T cells from infected ex-GF mice showed increased TNFα-secreting CD4 and CD8 T cells in the PP and PLN, respectively (Figures 6B,C and Supplementary Figures 9A,B). However, we did not observe any significant differences in IL-10-, IL-17a-, or IFNγ-secreting T cells in either the PP or PLN (Supplementary Figures 9C–H). We also found increased proportions of KLRG1-expressing CD8 T cells in virus-infected mice vs. the controls (Figure 6D and Supplementary Figure 9I), suggesting an increase in the antigen-experienced memory CD8 T cells. To test whether MNV4 infection affected the function of the Treg cells, we performed antigen-specific Treg suppression assays. We found that Tregs from MNV4-infected mice showed significantly greater suppression of the proliferation of BDC2.5 CD4+ T cells and secretion of IFNγ upon recognition of the antigenic peptide, compared to the Tregs from the uninfected controls (Figures 6E,F). Interestingly, we did not find any differences in IL10 or TGFβ production in the culture supernatants of the Treg suppression assays (Supplementary Figures 9J,K). To further confirm that MNV4, not the altered gut microbiota, was responsible for the Treg expansion, we colonized GF NOD mice with the gut bacteria from SPF NOD mice in the presence or absence of MNV4. We found that only when MNV4 was present was there an expansion of Tregs in the PLN and PP (Supplementary Figure 9L). Thus, our data suggest MNV4 predominantly promotes immune responses both in the intestinal tissue and the PLN, with the expansion and enhanced function of Treg cells in PLN, providing protection against T1D development in the infected mice.

### MNV4-Containing Fecal Filtrate Modulates T Cell Response Differently Dependent on Whether the T Cells Have Previously Been Exposed to MNV4

**Figure 7:**
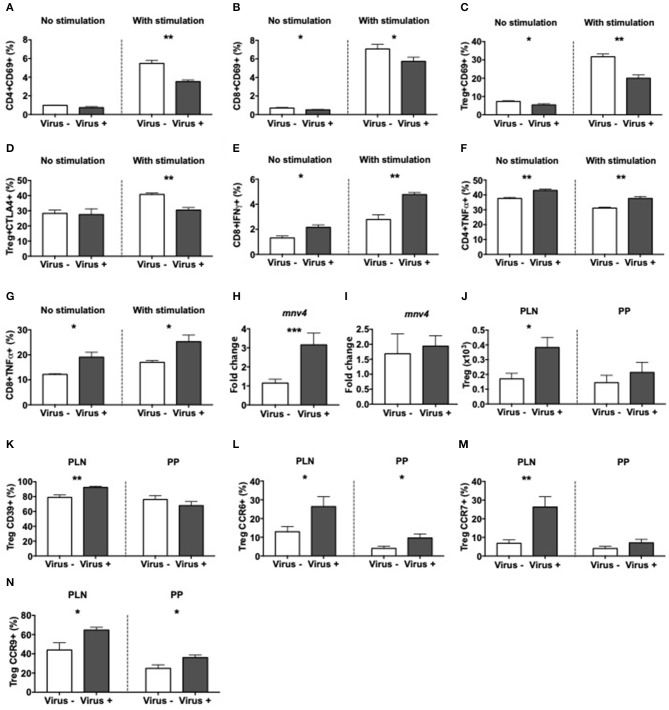
Altered T cell immunity after exposure to MNV4 *in vitro*. Fecal pellets from MNV4-infected 12-week old GF NOD were homogenized and filtrate was cultured with splenocytes from MNV4- or MNV4-colonized GF NOD mice. Cells were stimulated for 12 h prior to the addition of PMA, Ionomycin and GolgiPlug for a further 4 h preceding the surface and intracellular staining. As a control, splenocytes were not stimulated with MNV4-containing fecal filtrate but were stimulated with PMA, Ionomycin and GolgiPlug. **(A,B)** The proportion of CD69+ CD4 T cells **(A)** and CD8 T cells **(B)** were gated from live, single TCRbeta+CD19- cells prior to gating on CD4 or CD8 respectively and then CD69. **(C,D)** CD4+FoxP3+ Tregs were investigated for CD69+ **(C)** and CTLA4+ **(D)** cells. Tregs were gated as in B, prior to FoxP3 gating and then CD69 or CTLA4 gating. **(E–G)** The proportion of IFNγ-secreting CD8 T cells **(E)** and TNFα-secreting CD4 T cells **(F)** and CD8 T cells **(G)** were gated as in **A,B** prior to subsequent gating on IFNγ or TNFα. RNA from cultured T cells or APCs was isolated following exposure to UV-treated or UV-untreated (MNV4-containing) fecal material. Equal concentrations of cDNA were synthesized and then subjected to qPCR for MNV4 **(H,I)**. The relative expressions of these genes were determined using the 2^−ΔΔCT^ method by normalization with GAPDH. MNV4-exposed or MNV4-naive splenocytes were adoptively transferred into Rag-deficient NOD mice (4 × 10^6^/donor). Seven-days later mice were sacrificed and Tregs were investigated by flow cytometric analysis. Tregs were gated from live, single cell, CD4+TCRbeta+Foxp3+ T cells. Treg number **(J)** and the proportion of CD39- **(K)**, CCR6- **(L)**, CCR7- **(M)** and CCR9-expressing **(N)** Tregs are shown. All data were analyzed for significance using a Student's *T*-test. Data shown are representative of one of two experiments, averaged from experimental duplicates with *n* = 4 per group/experiment. ^*^P < 0.05, ^**^P < 0.01, ^***^p < 0.001.

To determine the response of immune cells to direct exposure to MNV4, splenocytes from norovirus+ and norovirus- ex-GF NOD mice were cultured overnight with the fecal filtrate collected from the ex-GF norovirus+ mice. There was minimal T cell activation (determined by CD69 expression) without exposure to the viral positive fecal filtrate regardless of the source of splenocytes (Figures 7A,B and Supplementary Figures 10A,B). However, upon exposure to the virus-positive fecal filtrate, T cells from MNV4 naive ex-GF mice were highly activated, whereas T cells from MNV4 infected ex-GF mice showed significantly reduced activation (Figures 7A,B and Supplementary Figures 10A,B). While we did not observe obvious changes in Tregs, we found that MNV4 exposure promoted more activation and increased expression of CTLA4 on Tregs in splenocytes from virus-naïve ex-GF mice compared to virus-experienced ex-GF mice (Figures 7C,D and Supplementary Figures 10C–E). This suggests that MNV could modulate Tregs. Again, there was no difference in IL-10- secreting CD4 T cells (Supplementary Figure 10F). Direct exposure of splenocytes to MNV-containing fecal filtrate induced significantly more IFNγ-secreting CD8 T cells and TNFα-secreting CD4 and CD8 T cells, respectively, from cells taken from MNV-experienced ex-GF mice compared to the cells from naïve ex-GF mice (Figures 7E–G and Supplementary Figures 10G–J). Interestingly, direct exposure of MNV-containing fecal filtrate did not have much effect on macrophages, dendritic cells or B cells from both MNV-experienced and MNV-naïve ex-GF mice (Supplementary Figure 11). To determine whether MNV4 infected T cells and/or APCs directly, we assessed MNV4 gene expression in purified splenic T cells and APCs by qPCR. Interestingly, we found that T cells but not APCs were infected by MNV4 (Figures 7H,I), suggesting that MNV has direct effect on T cells. Finally, to confirm whether exposure to MNV4 affected the Treg recruitment *in vivo*, we first cultured splenocytes from MNV4-naïve NOD mice *in vitro* in the presence or absence of MNV4 for 12 h, followed by adoptive transfer of the MNV-exposed T cells into Rag-deficient NOD mice. Treg expansion was observed in the recipients as early as a week after the transfer (Figure 7J). These Tregs, specifically in the PLN also expressed more CD39 (Figure 7K and Supplementary Figure 12A) when compared to the Tregs from the recipients that were transferred with non-MNV4-exposed cells. As CD39 positive Tregs have better suppressive function (51), this may explain the enhanced Treg suppression (Figure 6E) and protection from T1D (Figure 1A) observed in our study. Moreover, we demonstrated that the MNV4-exposed Tregs had increased expression of CCR6, CCR7, and CCR9, compared to the Tregs without exposure to MNV4 (Figures 7L–N, respectively, and Supplementary Figures 12B–D), however, only the increase in CCR7 expression was restricted to the PLN. This may suggest these Tregs are preferentially recruited via CCR7 to the pancreatic lymph nodes in response to chemokine expression (CCL19/CCL21).

## Discussion

Viral infections, particularly enteric viruses, have been linked to the development of T1D (12–15, 18, 20, 43, 52, 53); however, most of these studies have focused on the coxsackievirus or rotavirus, little is known the role of norovirus in T1D development. Using the NOD mouse model of human T1D, we investigated the effect of noroviral infection on the natural history of T1D development. We identified four novel findings in this study. First, unlike the studies in coxsackievirus or rotavirus, which promote T1D development (17, 54), norovirus infection protects mice from T1D development. Second, we discovered that norovirus infection induces an expansion of regulatory T cells but reduces proinflammatory T cells, specifically in pancreatic draining lymph nodes, potentially as a direct effect of viral infection. Third, we found that noroviral infection alters the composition of gut commensal bacteria in the hosts, which adds another plausible mechanism for T1D protection. This is different from an IL-10 deficient IBD mouse model, in which noroviral infection triggered gut microbiota-driven IBD development (35). Last but most importantly, we verified our findings by infection of GF NOD mice, which tested the viral effect more directly. Using GF mice, we identified that norovirus infection alters the phenotype of mucosal B cells and macrophage and inhibits mucosal IgG2a and IgG2b-producing B cells. Thus, our study demonstrated that noroviral infection protects NOD mice from T1D development, and this was associated with the expansion, recruitment and enhanced function of Treg cells, more evident in pancreatic draining lymph nodes, alteration of β-diversity in gut commensal bacteria and reshape mucosal B cells and macrophages.

Many studies including our own have shown that the gut microbiota can modify the immune system in NOD mice (2, 5, 8–11, 38, 40, 44, 55, 56) and the composition of gut microbiota is altered in individuals with pre-diabetes and human individuals with T1D (6, 7, 57–59). However, it is not clear if the effects are directly due to the gut commensal bacteria or enteric viruses, as many endogenous (bacteriophages) and exogenous (from the environment) viruses are present in the gut ecosystem. Furthermore, some enteric viruses are harmless whereas others are pathogenic, with the latter causing damage to the enterocytes and alteration of gut microbiota (60). It is also important to note that the gut microbiota composition can also alter the susceptibility of the virus to persist. Thus far, most of the studies in relation to viral infection and T1D development, in mouse and man, have been focused on the immunopathogenic effect; the knowledge of immune-regulatory effect of enteric viral infection is largely unknown. By probing the role of murine norovirus infection in T1D development in NOD mice, we discovered that MNV4 infection has beneficial effects in preventing T1D development in NOD mice. MNV4 infection promotes the expansion of Treg cells and the suppressive function of Tregs; and alters the mucosal B cell response. Further, MNV4 infection affected the overall richness of the gut bacteria and changed the β-diversity of the gut commensal bacteria. To distinguish virus-dependent gut commensal bacterial effects, we took a novel approach using GF NOD mice and reconstitute with a fecal filtrate preparation with or without norovirus, from which gut bacteria were removed. With this approach in GF mice, we are able to better identify the immune responses directly related to the noroviral infection. Importantly, we were able to recapitulate Treg expansion in infected ex-GF mice. Using a DSS induced colitis model, Kernbauer et al. showed that MNV infection replaced the immune-beneficial function of gut microbiota mediated by group 2 innate lymphoid cells (ILC2) and type 1 interferon signaling (61). Similarly, we found an increased gene expression of IL-25 in response to norovirus infection, which has been reported to induce and regulate ILC2 cells (62). We also found increased IL-4 in the intestinal wash of norovirus–infected mice, suggesting that there may be a role for IL-4-producing ILC2s in mediating some of the intestinal changes observed in our study. Although we did not find obvious changes in IL-5, IL-13, or Th2 cell markers, which are associated with ILC2 (62, 63), we did, however, observe changes in both the maturity of B cells and the proportion of follicular B cells in the PP, accompanied with alterations of IgG2 antibody production in the gut lumen but not in the circulation. Moreover, our results also demonstrated elevated soluble IFNα in the gut lumen and highly up-regulated genes encoded for IFNα receptor and Stat-1 (33) in the intestine tissue of norovirus infected ex-GF mice. This suggests that the norovirus induces a stronger local immune response.

It has recently reported that a strain of norovirus infects Tuft cells in the intestine (49). To determine whether MNV4 was present in IECs, we determined MNV4 gene expression by qPCR from isolated IECs of ex-GF mice. We found that MNV4 was highly expressed in the infected mice, suggesting that MNV4 can infect IECs. To investigate if MNV4 infection affects Tuft cells, we evaluated the Tuft cell markers (49) in the distal small intestine, by qPCR. We found enhanced expression of Tuft-cell related genes in our MNV4 infected mice, supporting the interactions of MNV-Tuft cell/IEC. It should be noted, however, that the tropism for MNV4 is not yet known, therefore, whether MNV4 directly infects Tuft cells, as does CR6 (49), or if the Tuft cells are indirectly affected by MNV4 infection remains to be determined.

In addition to Tuft cells, gut epithelial cells express an array of Toll-like receptors (TLRs). Noroviruses are single-stranded RNA (ssRNA) viruses and TLR7 and TLR8 are the receptors for ssRNA. Not surprisingly, both genes encoding TLR7 and TLR8 are highly up-regulated in the small intestinal tissue of the virally-infected ex-GF mice. Interestingly, gene expression of RIG-I, the receptor for double-stranded RNA (dsRNA), was also significantly up-regulated in the same tissue from the norovirus infected mice. It is possible that dsRNAs are derived from either replicating viral genomes or self-RNAs released upon either infection-mediated cell lysis or the physiological turnover of gut epithelial cells or a combination of all of the above.

As insulin-producing β-cell destruction is T cell mediated in T1D, norovirus infection will most likely impact the T cells directly or indirectly to affect the disease susceptibility. It is intriguing that MNV4 infection promotes the expansion of regulatory T cells *in vivo*, which in turn can suppress the ongoing β-cell destruction and prevent the mice from T1D development. This is supported by the reduction of proinflammatory cytokine-secreting T cells in the PLN of the infected mice. Interestingly, direct exposure of norovirus+ fecal material to splenocytes from naïve ex-GF mice resulted in a higher proportion of activated Tregs and increased CTLA-4 expression on Tregs, whereas the direct exposure of splenocytes from virally-experienced ex-GF mice to norovirus+ fecal filtrate induced fewer activated Tregs but more inflammatory T cells. We also observed that MNV4-exposed Tregs, following adoptive transfer into Rag-deficient NOD mice, expressed higher CD39 and were preferentially recruited to the PLN. This suggests that primary norovirus infection promotes an immune suppressive stage whereas secondary or persistent infection could lead to an inflammatory state. This may partially explain the rise of diabetes development after 20-weeks of age in the infected NOD mice as MNV4 actively replicates in the infected mice.

In our study we infected mice at ~4 weeks of age, leading to diabetes protection. It is possible that the age of the mice may influence either the ability of norovirus to modulate the immune system to promote tolerance over diabetogenicity or the type of host immune response to noroviral infection or both. In rotavirus studies, Graham et al. found that rotaviral infection in young NOD mice led to diabetes protection while infection in adulthood accelerated the development of T1D (16, 17). We will test this possibility in the future studies.

It is important to note that norovirus is a common mouse pathogen in animal facilities, causing persistent infections (64). Eradication of MNV from animal facilities requires rederivation in addition to strict observance of stringent standard operating protocols (65–67). Our study shows that MNV can protect NOD mice from developing T1D; however, it is possible that MNV infection may result in negative effects in different disease models using different mouse strains. Thus, it is recommended that more specific pathogens should be tested and reported in research studies.

In summary, our findings demonstrate that norovirus infection promoted protection from T1D development in NOD mice by increasing the number of Tregs, increasing intestinal immunity (including Tuft cells), changing the composition of gut commensal bacteria and local immune cell phenotype and function. Given the high virulence of norovirus in humans, it is likely to be encountered by individuals at risk of developing T1D, particularly as children are more susceptible. Our study not only highlights the importance of virus-commensal bacteria-immune interactions in T1D but also suggests that some enteric viral infections may be beneficial in preventing autoimmune disease such as type 1 diabetes.

## Data Availability Statement

All relevant data are contained within the manuscript and the raw sequencing data are available on request.

## Ethics Statement

The animal study was reviewed and approved by Institutional Animal Care and Use Committee at Yale University.

## Author Contributions

JP, NT, DE-A, JP, YH, KH, and SC conducted experiments. JP, NT, DE-A, YH, PS, and LW designed the experiments and analyzed the results. FW consulted the study. JP, NT, DE-A, and LW wrote the manuscript. FW and SC edited the manuscript. PS and LW conceived and supervised the study.

### Conflict of Interest

The authors declare that the research was conducted in the absence of any commercial or financial relationships that could be construed as a potential conflict of interest.
