# Supplementary figures and images for: Norovirus Changes Susceptibility to Type 1 Diabetes by Altering Intestinal Microbiota and Immune Cell Functions
Source: Front Immunol. 2019 Nov 12;10:2654. doi: 10.3389/fimmu.2019.02654 (PMC6863139; doi:10.3389/fimmu.2019.02654)

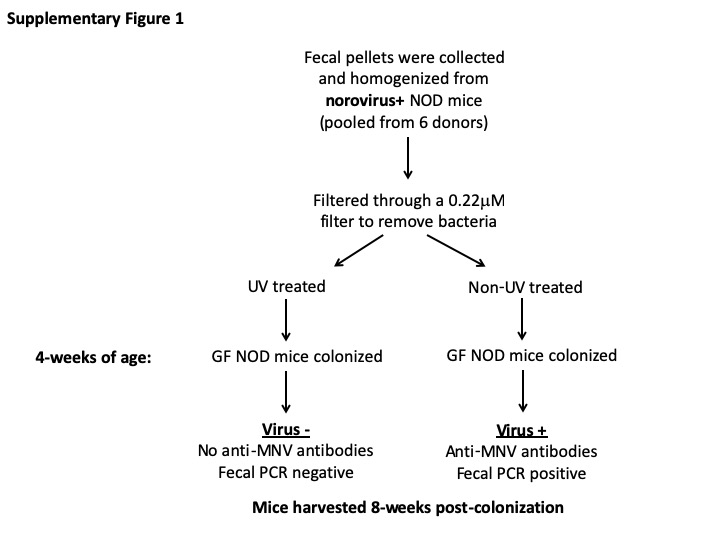

Supplement: Supplementary file 1 [file Image_1.JPEG]

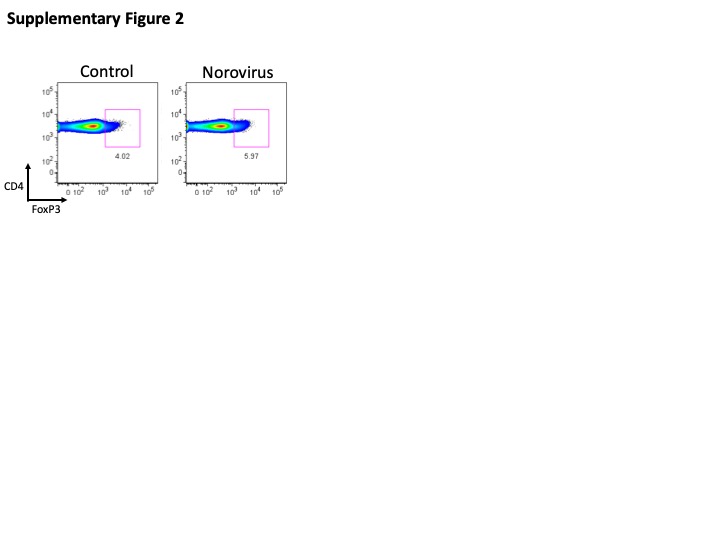

Supplement: Supplementary file 2 [file Image_2.JPEG]

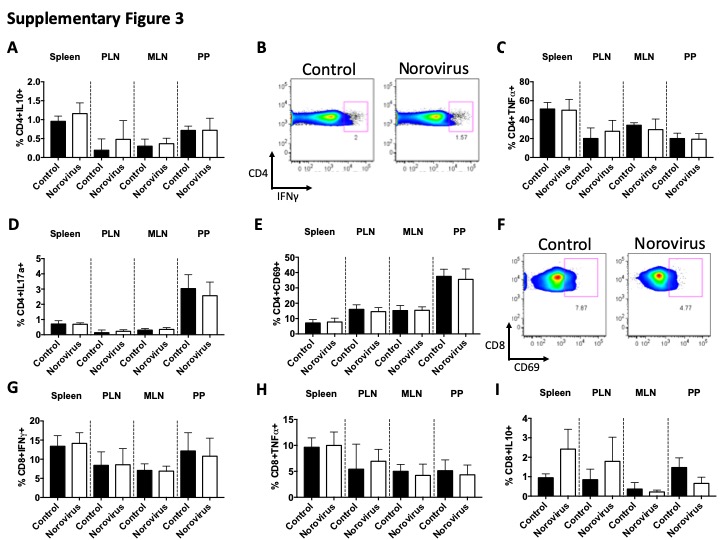

Supplement: Supplementary file 3 [file Image_3.JPEG]

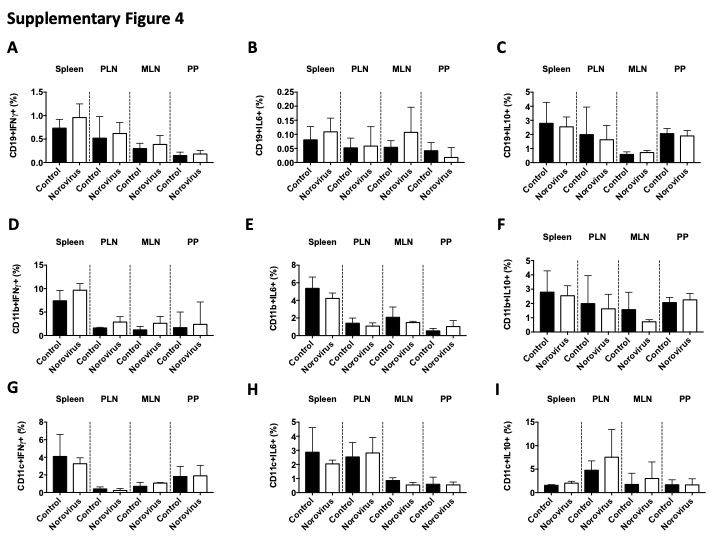

Supplement: Supplementary file 4 [file Image_4.JPEG]

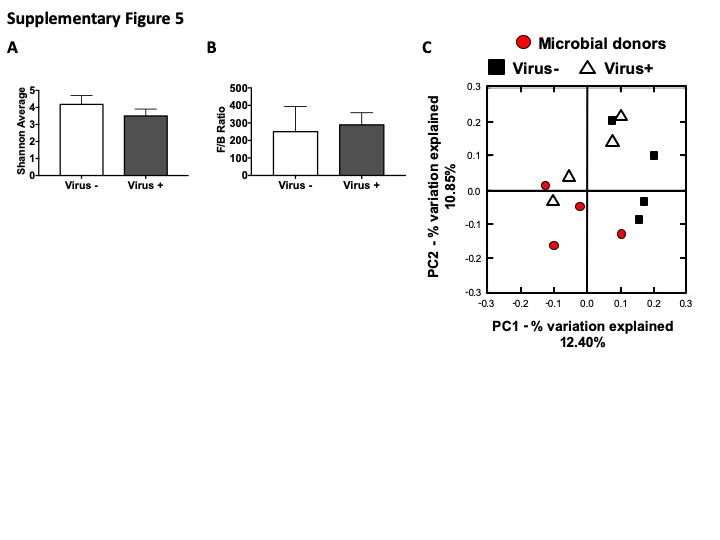

Supplement: Supplementary file 5 [file Image_5.JPEG]

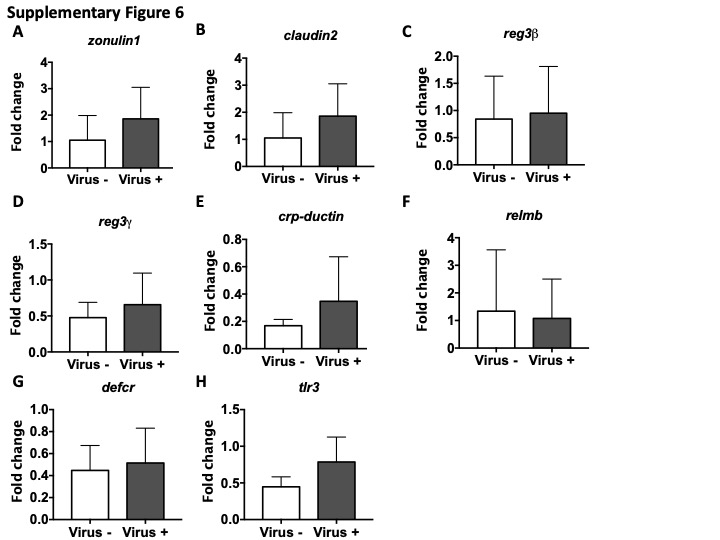

Supplement: Supplementary file 6 [file Image_6.JPEG]

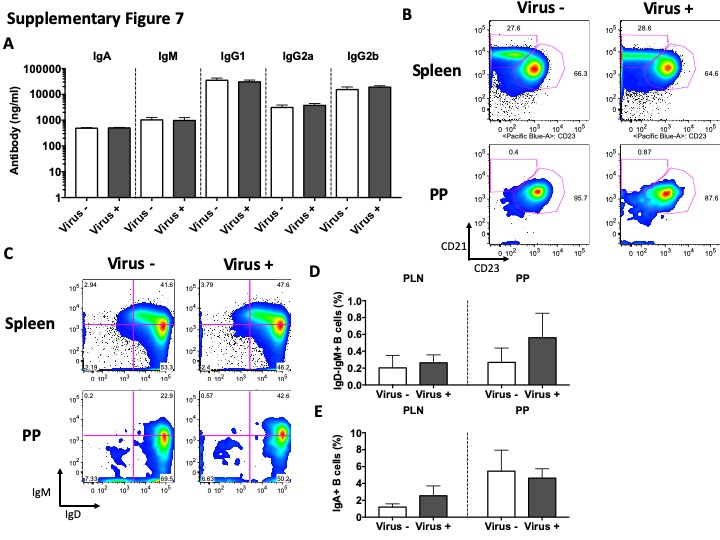

Supplement: Supplementary file 7 [file Image_7.JPEG]

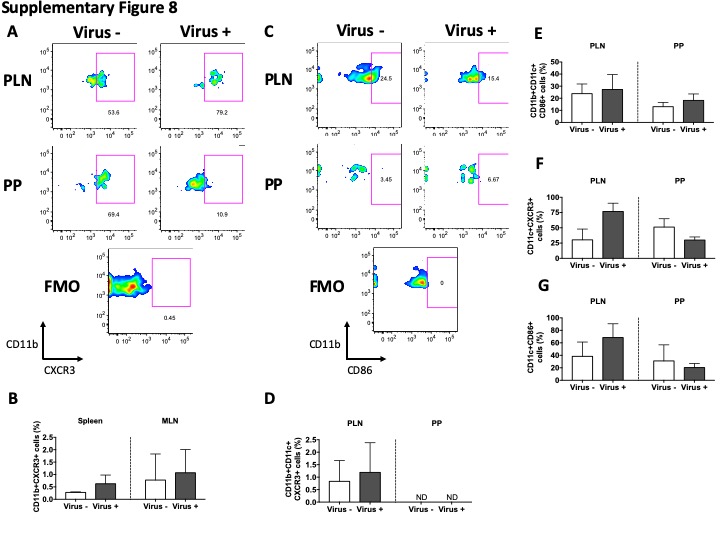

Supplement: Supplementary file 8 [file Image_8.JPEG]

**Supplementary Figure 9**

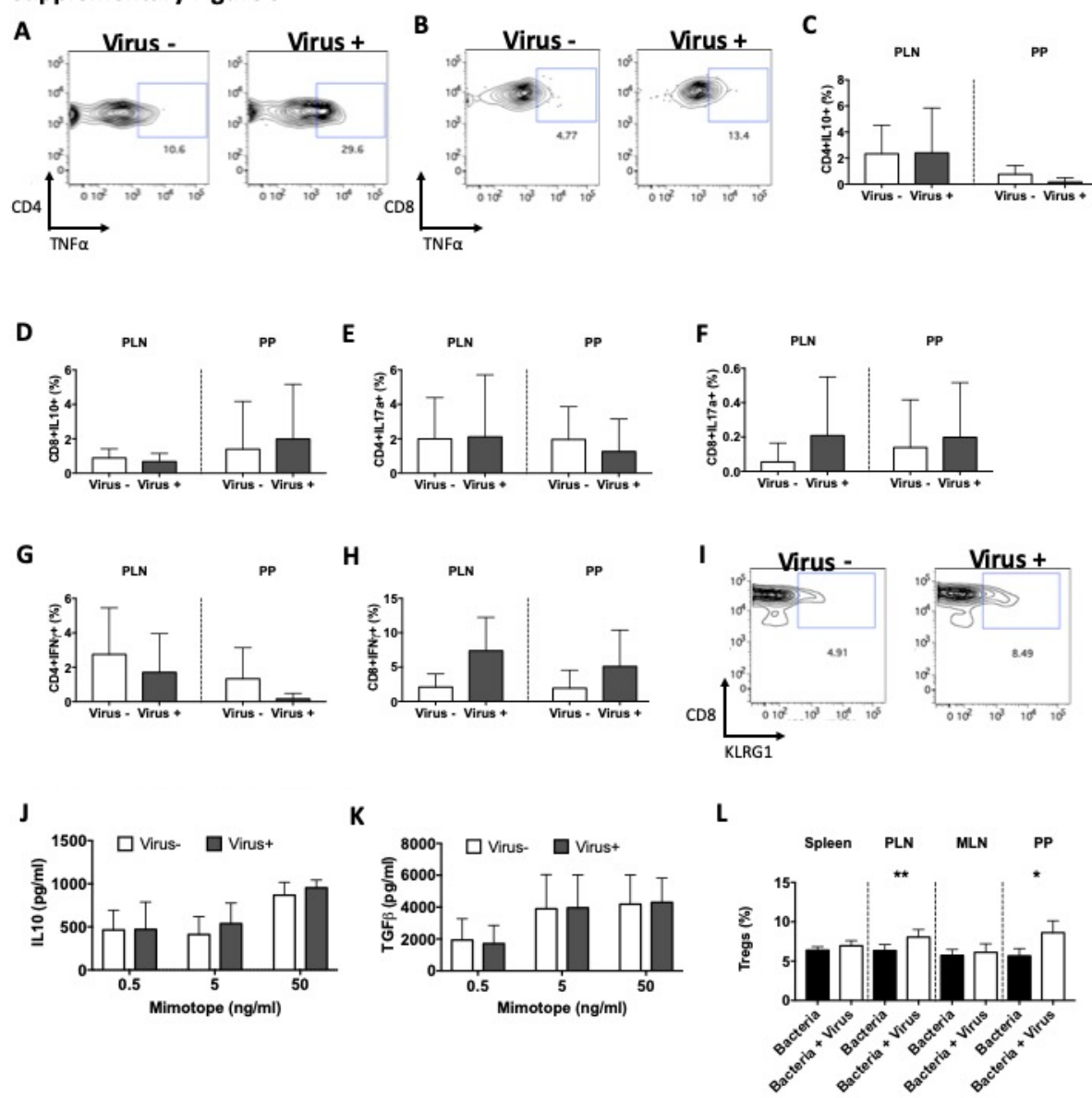

Supplement: Supplementary file 9 [file Image_9.pdf]

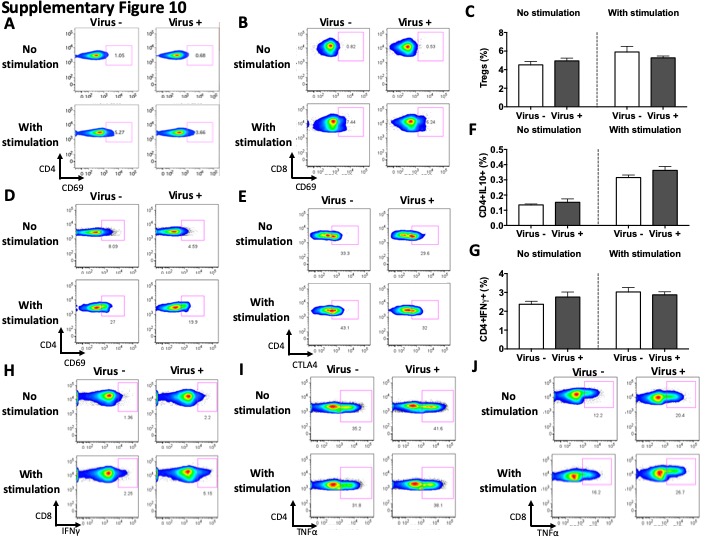

Supplement: Supplementary file 10 [file Image_10.jpeg]

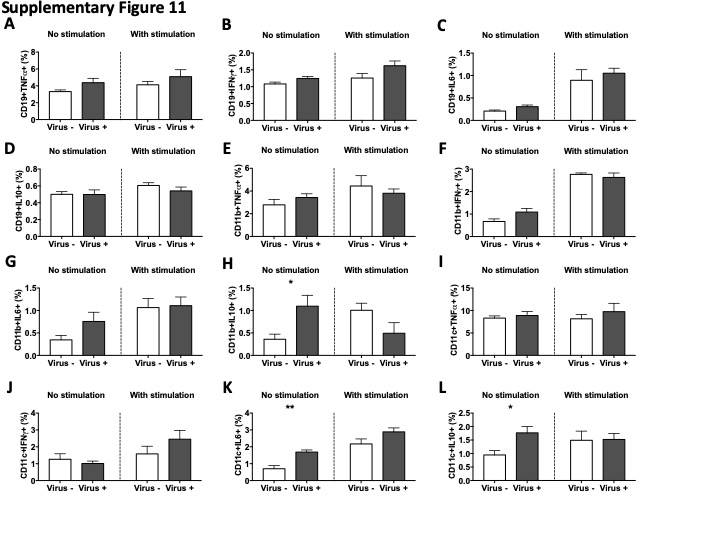

Supplement: Supplementary file 11 [file Image_11.jpeg]

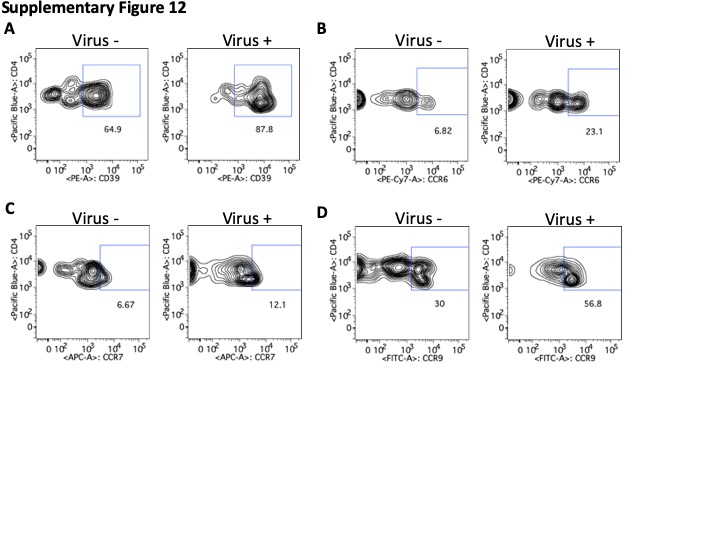

Supplement: Supplementary file 12 [file Image_12.jpeg]
