# Supplementary material for: Norovirus Changes Susceptibility to Type 1 Diabetes by Altering Intestinal Microbiota and Immune Cell Functions
Source: Front Immunol. 2019 Nov 12;10:2654. doi: 10.3389/fimmu.2019.02654 (PMC6863139; doi:10.3389/fimmu.2019.02654)
Supplement: Supplementary file 13 [file Data_Sheet_1.doc]

Supplementary Material

**Supplementary Table 1 – qPCR primer list**

| **Gene** | **Forward (5’-3’)** | **Reverse (5’-3’)** |
| --- | --- | --- |
| **Mnv5033** | GGAACGCTCAGCAGTCTTTG | CAAGAAGAGGGAGTTGAATG |
| **dclk1** | TGAACAAGAAGACGGCTCACTCC | GCTGGTGGGTGATGGACTTGG |
| **il25** | ACAGGGACTTGAATCGGGTC | TGGTAAAGTGGGACGGAGTTG |
| **succinate receptor** | GGGGACCTATGGAGATGTCT | GCCAGCGAGATTAAAATGGCAA |
| **tlr7** | ATGTGGACACGGAAGAGACAA | GGTAAGGGTAAGATTGGTGGTG |
| **tlr8** | ACAATGCTCCATTTCCTTGC | CTGAGGGAAGTGCTGGAAAG |
| **tlr3** | GTGAGATACAACGTAGCTGACTG | TCCTGCCATCCAAGATAGCAAGT |
| **ifnar1** | GGAGGGAGAATGTGTTTTTGAG | TCCAGACTACGCACTGTGTCTT |
| **ifn**λ | AGCTGCAGGTCCAAGAGCG | GGTGGTCAGGGCTGAGTCATT |
| **rig1** | GCTGTGTGGAAAGTGCAAGA | AGTTCTGTTTGGCGCAGAAT |
| **stat1** | CTGAATATTTCCCTCCTGGG | TCCCGTACAGATGTCCATGAT |
| **nfκb** | GGTATGGCTACTCGAACTACGG | TTTCCTTCTCAGGGAGAGTCAG |
| **irf1** | CCTGCTTTGTATCGGCCTGT | ATGCCAATCACTCGAATGCG |
| **irf3** | GAGAGCCGAACGAGGTTCAG | CTTCCAGGTTGACACGTCCG |
| **zonulin1** | CACCGGAGTGATGGTTTTCT | CCACCTCTGTCCAGCTCTTC |
| **claudin2** | TCATGCCCACCACAGAGATA | TATGTTGGTGCCAGCATTGT |
| **reg3β** | CTGCCTTAGACCGTGCTTTC | CCCTTGTCCATGATGCTCTT |
| **reg3γ** | TTCCTGTCCTCCATGATCAAAA | CATCCACCTCTGTTGGGTTCA |
| **crp-ductin** | TGAACCGTGTGACAGTGGTCTTCA | TCTCCTTGTCACACTGCCATCTGT |
| **relmb** | AGCTCTCAGTCGTCAAGAGCCTAA | CACAAGCACATCCAGTGACAACCA |
| **defcr** | ATCATCCAGGTGATTCCCAGCCAT | TTCCGGGTCTCCAAAGGAAACAGA |
| **gapdh** | TGACATCAAGAAGGTGGTGAAG | TGCTGTAGCCGTATTCATTGTC |

**Supplementary Figure Legends**

**Supplementary Figure 1 –Experimental design of colonizing germ-free (GF) mice with gut microbiota with or without MNV**

Fecal microbial suspensions (containing norovirus) from norovirus+ NOD mice were pooled and divided into 2 portions. Half the microbial solution was exposed to UV-treatment (destroying the virus; designated virus-) and the other half remained non-exposed to UV-treatment (virus+). GF NOD mice were subsequently gavaged with these preparations. Longitudinal fecal and blood samples were collected from the colonized GF mice to determine the presence of MNV in the feces and blood. The colonized GF NOD mice were terminated 8-weeks later for study.

**Supplementary Figure 2 – Treg gating example**

CD4+FoxP3+ T cells were investigated from the spleen, pancreatic lymph node (PLN), mesenteric lymph node (MLN) and Peyer’s patches (PP) of norovirus-free and norovirus-infected NOD mice. A representative example of a FoxP3+ Treg gating plot from the PLN after first gating on live single TCRbeta+CD4+CD8- T cells is shown.

**Supplementary Figure 3 – Cytokine secreting T cell proportions**

Cells from the spleen, pancreatic lymph node (PLN), mesenteric lymph nodes (MLN) and Peyer’s patches (PP) were stimulated for 4 hours in the presence of PMA, Ionomycin and Golgi Plug prior to surface and intracellular staining. (A) The proportion of IL10-secreting CD4 T cells gated from live, single CD4+TCRbeta+CD8- T cells. (B) Representative gating plots of CD4 IFNγ-secreting T cells from the PLN. (C-D) The proportion of TNFα- (C) and IL17a-secreting CD4 T cells (D) gated as in A. (E) CD69 expression was assessed in CD4 T cells directly ex vivo without stimulation using the same gating strategy as in A. (F) Representative flow gating plot of CD69+ CD8 T cells directly ex vivo after gating on live, single CD8+TCRbeta+CD4- T cells. (G-I) The proportion of IFNγ- (G), TNFα- (H) and IL10-secreting CD8 T cells (I) post 4-hour stimulation in the presence of PMA, Ionomycin and Golgi Plug, gated as in F. Data were analyzed for significance using a Student’s T test. Data shown in the figures are pooled from 2 independent experiments (n=9-11).

**Supplementary Figure 4 – Frequency of cytokine-secreting APC**

Cells from the spleen, pancreatic lymph node (PLN), mesenteric lymph nodes (MLN) and Peyer’s patches (PP) were stimulated for 4-hours in the presence of PMA, Ionomycin and Golgi Plug prior to surface and intracellular staining. (A-C) The proportion of IFNγ-, IL-6- and IL10-secreting B cells after gating from live, single TCRbeta- cells. (D-F) The proportion of IFNγ-, IL-6- and IL10-secreting CD11b+ macrophages after gating from live, single TCRbeta-CD19-MHCII+CD11c- cells. (G-I) The proportion of IFNγ-, IL-6- and IL10-secreting CD11c+ dendritic cells gated from live, single TCRbeta-CD19-MHCII+CD11b- cells. Data were analyzed for significance using a Student’s T test. Data are shown from 2 pooled independent experiments (n=9-11).

**Supplementary Figure 5 – Similar composition of gut microbiota in ex-GF NOD mice colonized with gut bacteria with or without norovirus**

Fecal pellets were collected from 12-week old ex-GF NOD mice that were MNV-free (virus-) or MNV-infected (virus+) and sequenced. (A) α-diversity was assessed using the Shannon diversity index. (B) The Firmicutes/Bacteroidetes (F/B) ratio were calculated from the phylogenetic information based on the sequencing results. (C) β-diversity is represented by a principal component analysis plot and no significant microbial differences were found by a multiple T-test with FDR correction. Microbial donors are also shown for comparison (red circles). Data were assessed for significance using a Student’s T-test (A-B). Data are representative of one of two experiments (n=4 mice/experiment).

**Supplementary Figure 6 – Gene expression profile in intestine following infection of GF NOD mice with MNV4**

GF NOD mice were orally gavaged at ~4 weeks of age with virus- or virus+ fecal filtrate preparations as illustrated in Supplementary Fig. 1 (A-H) RNA from the distal small intestine, immediately adjacent to the cecum, was extracted and equal concentrations of cDNA synthesized. cDNA was then used for qPCR for genes associated with gut permeability (zonulin1 and claudin2; A-B), antimicrobial peptides (C-G) and Toll-like receptor 3 (tlr3; H). The relative expression of these genes was determined using the 2^-ΔΔCT^ method by normalization with GAPDH. Student’s T-test was used for statistical analysis. Data shown are from one of two experiments with n=4 per group/experiment.

**Supplementary Figure 7 – Characterization of B cells after infection of GF NOD mice with MNV4**

GF NOD mice were orally gavaged at ~4 weeks of age with virus- or virus+ fecal filtrate preparations as illustrated in Supplementary Fig. 1. Viral infection was confirmed by PCR on fecal samples and the presence of anti-MNV specific antibodies in the serum samples. (A) Levels of different isotype of serum immunoglobulins were determined by ELISA when the mice were terminated. (B-E) The proportion of different B cell subsets was determined by flow cytometric analysis. All the cells were gated from live, single CD19+TCRbeta- cells. (B-C) Representative flow cytometric gating plots showing CD21 and CD23 gating from the spleen and Peyer’s patches (PP) (B) and IgM and IgD gating (C). (D) Summary of IgM+IgD- B cells gated as shown in C from the pancreatic lymph nodes (PLN) and PP. (E) Summary of IgA+ B cells gated as in B, prior to gating on IgA. Student’s T-test was used for statistical analysis. Data shown in the figure are from one of two experiments with n=4 per group/experiment.

**Supplementary Figure 8 – Characterization of macrophages and dendritic cells following infection of GF NOD mice with MNV4**

GF NOD mice were orally gavaged at ~4 weeks of age with virus- or virus+ fecal filtrate preparations as illustrated in Supplementary Fig. 1. Viral infection was confirmed by PCR on fecal samples and the presence of anti-MNV specific antibodies in the serum samples. The proportion of cell subsets was determined by flow cytometry analysis. (A) Representative flow cytometric gating plots showing CXCR3 gating of macrophages gated from live, single CD19-TCRbeta-IA^g7^(MHCII)+CD11b+CD11c- cells. (B) The proportion of CXCR3+ macrophages from the spleen and MLN. (C) Representative flow cytometric gating plots showing CD86 gating of macrophages gated from live, single CD19-TCRbeta-IA^g7^(MHCII)+CD11b+CD11c- cells. (D-G) The proportion of CXCR3+ and CD86+ CD11b+CD11c+ cells (D-E respectively) or CD11b-CD11c+ dendritic cells (F-G respectively) gated from live, single CD19-TCRbeta-IA^g7^(MHCII)+ cells prior to gating on CXCR3 and CD86. Student’s T-test was used for statistical analysis. Data shown are from one of two experiments with n=4 per group/experiment.

**Supplementary Figure 9 – Representative T cell gating plots and summary of cytokine- secreting T cells**

GF NOD mice were orally gavaged at ~4 weeks of age with virus- or virus+ fecal filtrate preparations as illustrated in Supplementary Fig. 1. Viral infection was confirmed by PCR on fecal samples and the presence of anti-MNV specific antibodies in the serum samples. (A-B) Representative gating plots of TNFα-secreting CD4 T cells (A) and CD8 T cells (B) after gating from live, single TCRbeta+CD19- cells. (C-H) Frequency of IL10-, IL17a- and IFNγ-secreting CD4 and CD8 T cells after gating from live, single TCRbeta+ cells. (I) Representative gating plots of KLRG1+ CD8 T cells after gating from live, single TCRbeta+CD19-CD4- cells. (J-K) IL10 and TGFβ were assessed by ELISA from the culture supernatants of the Treg suppression assay. (L) Summary of Treg proportions in GF NOD mice that were orally gavaged at ~4 weeks of age with pelleted fecal microbiota devoid of MNV4 (bacteria) or microbiota containing MNV4 (bacteria + virus) from norovirus-infected NOD mice. Student’s T-test was used for statistical analysis. Data shown from one of two experiments. *P<0.05, **P<0.01.

**Supplementary Figure 10 – Representative cell gating and characterization of T cell stimulated with MNV4 *in vitro***

Splenocytes from MNV4-naive or MNV4-infected GF NOD mice were stimulated for 12 hours with filtrate of fecal pellets from MNV4-infected 12-week-old ex-GF NOD and followed by addition of PMA, Ionomycin and GolgiPlug for a further 4-hour stimulation, prior to surface and intracellular staining. Splenocytes stimulated with MNV4-negative filtrate followed by PMA, Ionomycin and GolgiPlug were used as controls. (A-B) Representative flow cytometric gating plots of CD69+ CD4 T cells (A) and CD8 T cells (B), after gating from live, single TCRbeta+CD19- cells. (C) The summary of Treg frequency with the same gating strategy as described in Supplementary Fig. 2 legend. (D-E) Representative gating plots of CD69+ and CTLA4+ Tregs. (F-G) Summary of IL10- and IFNγ-secreting CD4 T cell proportions with the same gating strategy as described above. (H-J) Representative flow cytometric gating plots of IFNγ–secreting CD8 T cells (H) and TNFα-secreting CD4 (I) and CD8 (J) T cells. Student’s T-test was used for statistical analysis. Data shown are from one of two experiments, n=4 per group/experiment.

**Supplementary Figure 11 – Characterization of APC after stimulation with MNV4+ fecal filtrate *in vitro***

Splenocytes from MNV4-naive or MNV4-infected GF NOD mice were stimulated for 12 hours with filtrates of homogenized fecal pellets from MNV4-infected 12-week-old GF NOD, followed by the addition of PMA, Ionomycin and GolgiPlug for a further 4 hour culture before the surface and intracellular staining. Splenocytes stimulated with MNV4-negative filtrate followed by PMA, Ionomycin and GolgiPlug were used as controls. (A-D) Summary of the proportion of TNFα-, IFNγ-, IL-6- and IL10-secreting B cells gated from live, single TCRbeta- cells. (E-H) Summary of the proportion of TNFα-, IFNγ-, IL-6- and IL10-secreting CD11b+ macrophages gated from live, single TCRbeta-CD19-MHCII+CD11c- cells. (I-L) Summary of the proportion of TNFα-, IFNγ-, IL-6- and IL10-secreting CD11c+ dendritic cells gated from live, single TCRbeta-CD19-MHCII+CD11b- cells. Student’s T-test was used for statistical analysis and the data shown are from one of two experiments, n=4 per group/experiment. *p<0.05

**Supplementary Figure 12 – Expression of CD39 and chemokine receptors on Tregs**

Splenocytes from MNV4-naive GF NOD mice were stimulated for 12 hours with filtrates of homogenized fecal pellets from MNV4-infected 12-week old GF NOD. After washing, the cells were adoptively transferred into SPF Rag-deficient NOD mice (4x10^6^/mouse). Pancreatic lymph nodes were harvested from the recipients 7 days post-transfer and assessed for the expression of CD39 and chemokine receptor on Tregs. The expression of CD39 (A) was analyzed after gating CD4+CD25+FoxP3+ cells from live, single cells, and the expression of CCR6 (B), CCR7 (C) or CCR9 (D) on Tregs was analyzed using the same gating strategy. All the gating was controlled with an isotype control antibody.
